# Extremophiles and biotechnology: current uses and prospects

**DOI:** 10.12688/f1000research.7432.1

**Published:** 2016-03-24

**Authors:** James A. Coker

**Affiliations:** 1 Department of Biotechnology, University of Maryland, Adelphi, MD, USA

**Keywords:** Extremophiles, Biotechnology, biofuels, Carotenoids, Proteases, Lipases

## Abstract

Biotechnology has almost unlimited potential to change our lives in very exciting ways. Many of the chemical reactions that produce these products can be fully optimized by performing them at extremes of temperature, pressure, salinity, and pH for efficient and cost-effective outcomes. Fortunately, there are many organisms (extremophiles) that thrive in extreme environments found in nature and offer an excellent source of replacement enzymes in lieu of mesophilic ones currently used in these processes. In this review, I discuss the current uses and some potential new applications of extremophiles and their products, including enzymes, in biotechnology.

## Introduction

The impact of biotechnology on our lives is inescapable. Some of these impacts are well publicized, like the process of generating biofuels. However, there are numerous other applications that are not widely known outside of specialist circles that affect our daily life, such as food and drink (e.g. lactose-free milk ^1^ and bioinsecticides ^2^), how we make and wash our clothes (e.g. cellulases to produce ‘stone-washed’ jeans ^3^, lipases ^4^, and proteases ^5^ in detergents), and the medications we take to remain healthy, just to name a few examples.

Many of the reactions performed in the process of making these products are often not optimized because mesophilic enzymes are used at extremes of temperature, pressure, salinity, and pH ^6^. The efficiency of these enzymes is often improved through genetic and/or chemical modification ^7, 8^ as well as immobilization strategies ^9^, all of which are designed to produce biocatalysts with improved properties such as increased activity and/or stability to use in specific industrial processes. This can be a lengthy and (more importantly) costly enterprise, especially since nature provides many readily available alternatives in the form of extremozymes, which are found in organisms that thrive in extremes of temperature (as high as 122°C and as low as −12°C), pressure (as high as 1000 atm), salinity (up to and including saturating levels), and pH (from 0 to 6 and 8 to 12) ^10– 15^. As such, their enzymes are already adapted to work under the extreme conditions of many industrial processes.

To date, few extremophiles/extremozymes have found their way into large-scale use in the field of biotechnology ^16^; however, their potential is undeniable in many applications. Four success stories are the thermostable DNA polymerases used in the polymerase chain reaction (PCR) ^17^, various enzymes used in the process of making biofuels ^18^, organisms used in the mining process ^19^, and carotenoids used in the food and cosmetic industries ^20^. Other potential applications include making lactose-free milk ^1^; the production of antibiotics, anticancer, and antifungal drugs ^6^; and the production of electricity or, more accurately, the leaching of electrons to generate current that can be used or stored ^21^.

This review initially focuses on the four success stories of extremozymes in biotechnology. Then it discusses the most prominent sectors of the enzyme market (glycosyl hydrolases, lipases, proteases, and those of medical importance) where the application of extremophiles/extremozymes could replace currently used enzymes to make reactions more efficient and/or cost effective.

## DNA polymerases

It is difficult to overstate the success or impact the DNA polymerases from the thermophiles *Thermus aquaticus*, *Pyrococcus furiosus*, and *Thermococcus litoralis*, otherwise known as Taq ^22^, Pfu ^23^, and Vent ^24^, respectively, have had in biotechnology. Without a doubt, the automated version of the PCR would not have been possible without these enzymes. During its patent lifetime, PCR earned its rights holders over $2 billion in royalties ^25^. It is difficult to imagine our lives without PCR, especially for a typical bench scientist. However, fields once thought to be far removed from science, like law enforcement, have also benefitted greatly from PCR by making it more readily possible to identify and rule out suspects on the basis of their DNA profile ^26^. Even the entertainment industry, with its many movies, novels, and TV shows centered on forensic science and PCR-based technologies, has greatly benefitted from PCR. The impact these three hyperthermophilic enzymes have had on biotechnology and our current culture can only be described as immense.

## Biofuel production

In an effort to supplement the planet’s dwindling supply of fossil fuels, there has been a concerted effort to generate similar fuels using biomass (e.g. corn, beets, wheat, and sugar cane). Depending on the specific source, biofuels can be categorized into first and second generation. First-generation biofuels are those derived from the ‘easily’ hydrolyzed sugars, starches, and oils of available crops, whereas second-generation biofuels are derived from lignocellulosic material, which is more resistant to hydrolysis.

Biofuels can also be categorized by the eventual end products: butanol, ethanol, hydrogen, methane, and biodiesel. Traditional methods of biobutanol and bioethanol production involve the use of a chemical process supplemented with the use of mesophilic microorganisms such as *Saccharomyces cerevisiae* and *Clostridium* species ^27^. The production of hydrogen traditionally relies on a chemical/catalyst process ^28^; however, larger-scale microorganism-based systems using the thermophiles *Caldicellulosiruptor saccharolyticus* and *Thermotoga elfii* have been developed recently ^29^. In contrast to the other products, methane has always been produced using a consortium of microorganisms, which include methanogens (extremophiles that are the only known biologic producers of methane) ^18^.

Many of the steps in biofuel production involve high temperatures and extremes of pH; therefore, extremophiles are ideal candidates to replace the mesophilic organisms used in traditional methods. For example, *Thermoanaerobacterium saccharolyticum* is able to utilize hemicellulose and pentose sugars like xylose as a starting material to produce ethanol ^30^. Engineered versions of this thermophile have shown great promise in producing large quantities of ethanol and minimizing other side reactions/products ^31^. There are also numerous applications for extremophiles in the production of hydrogen through anaerobic fermentation and hydrogenases. The use of strains of *Caldicellulosiruptor*, *Thermoanaerobacterium* ^32^, *Pyrococcus* ^33^, and *Aeropyrum* ^34^ shows immense potential. The research is still very preliminary; however, recent advances such as the engineering of a hyperthermophile are quite promising ^35, 36^.

The two products produced by microorganisms that show the most commercial success are biodiesel and butanol. Biodiesel harvests the power of high lipid content (>75% dry weight) algae, most of which contain long-chain hydrocarbons like those found in petroleum. There are several extremophilic algae (e.g. *Cyanidium caldarium* ^37^ and *Galdieria sulphuraria* ^38^) that meet these requirements. Engineered halophilic algae also hold great promise, as they can be grown in open containers since the high salinity required for their growth inhibits other microbes. This means they can be grown in underutilized environments such as the oceans and arid/desert environments ^39^.

Butanol is quite inhibiting to the growth of microorganisms compared with ethanol (most organisms cannot tolerate more than 2%). As such, organisms need to be modified in order to overcome product inhibition and withstand large quantities of butanol. Currently, Green Biologics is producing biobutanol from corn stock by using thermophilic *Clostridium*. Other companies such as Gevo, Joule Unlimited, and Solazyme are also able to produce large-scale volumes of bioethanol and biodiesel as well as jet fuel for both civilian and military use. Additionally, Sapphire Energy has moved one step back in the process and generates what it calls ‘Green Crude’, which can act as a replacement for crude oil in the existing petroleum infrastructure.

## Biomining

In addition to biofuels, another important application of extremophiles and their enzymes can be found in the mining sector ^40^. This process, also known as bioleaching, is the removal of insoluble metal sulfides or oxides by using microorganisms ^41^. It is a safer and more environmentally friendly way to extract metals compared with traditional heap leaching, which involves the use of several chemicals, including cyanide, to bind and separate specific minerals/metals from others.

In 1992, biomining accounted for 10% of worldwide copper production ^6^, but current estimates place it at around 15% for copper and 5% for gold ^42^. Extraction rates are around 90% from biomining compared with 60% for traditional heap leaching ^41^. Biomining techniques have successfully been employed to mine metals such as gold, silver, copper, zinc, nickel, and uranium. The organisms used in this process are acidophiles such as *Acidithiobacillus* and *Ferroplasma*. However, depending on the conditions, more thermophilic strains, like *Sulfolobus* and *Metallosphaera* ^40, 41^, may have to be employed.

Although biomining is generally safe, it does need to be tightly controlled, since it can result in acid mine drainage (AMD), which occurs when acidic water, generated by the oxidation of sulfides from the mine, begins flowing or leaching out of the mine. Since the acidophiles employed in biomining thrive in acidic and usually heavy-metal environments, AMD results in an environment that is not only very acidic but also rich in heavy metals. Copper, zinc, and nickel mines are the most common sources of AMD ^41^. Interestingly, mesophilic and sometimes psychrophilic acidophiles are the main culprits of AMD ^41^. However, when thermophiles are used in biomining, the possibilities of AMD are reduced and costs associated with the cooling of processing tanks are kept to a minimum.

## Carotenoids

Carotenoids are natural pigments and in extremophiles are most often associated with the halophilic archaea and algae ^43^. Most carotenoids cannot be synthesized/extracted from organisms at levels that are useful for industry; however, there are three exceptions to this: bacteriorhodopsin, canthaxanthin, and β-carotene ^44^.

Bacteriorhodopsin is a membrane-bound retinal pigment as well as a proton pump that functions as a rudimentary form of photosynthesis. It is a very stable molecule and harvested from the extreme halophilic archaeon *Halobacterium salinarum* ^43^. It has been adapted for use in a wide range of applications from holography, artificial retinas, photochromic dyes, spatial light modulators, and the renewal of biochemical energy ^45^.

Canthaxanthin is a lipid-soluble antioxidant used as a food dye and a feed additive. As a feed additive, it is used in fish, crustacean, and poultry farms. It is also used in the cosmetics industry and usually is the primary ingredient in tanning pills ^46^. As with bacteriorhodopsin, halophilic archaea are the producers of choice with *Haloferax alexandrinus* being the preferred strain ^47^.

β-carotene is a red/orange pigment and the primary colorant in carrots, pumpkins, and halophilic microorganisms. The halophilic alga *Dunaliella salina* is the major source for β-carotene, as its commercial-scale growth results in 30–40 g dry weight/m ^2^ per day ^39^. Due to its chemical nature, it is a lipid/oil- and water-soluble molecule, which makes it excellent as an additive in the baking process (e.g. food coloring) and emulsions (e.g. confectionery and prepared foods). However, its primary use is probably as a food supplement ^39^.

## Proteases/lipases

Proteases and lipases, combined with the gylcosyl hydrolases, account for more than 70% of all enzymes sold ^48^ while proteases alone are the most widely used class of enzyme. Proteases have numerous applications in diverse fields; however, the largest application is in laundry detergents, where they have been a standard component since 1985 and are used to break apart and remove protein-based stains ^49^. The other major uses for proteases are in the fields of cheese making, brewing, and baking. Typically, the microbial proteases used are mesophilic and derived from *Bacillus* species and produced by companies such as Novozymes and Genencor. However, explorations using psychrophilic proteases to enhance cold water washing have taken place. Unfortunately, most psychrophilic enzymes have proven to be unusable due to low stability at room temperature. However, through directed evolution, a chimeric psychrophilic/mesophilic protease was generated that improved performance during cold water washing ^50^.

Lipases are a billion-dollar industry ^51^ and very attractive for use in industrial settings because of their broad range of substrates, high degree of specificity, and stability ^52^. Although their applications in laundry detergents (i.e. low temperatures and alkaline conditions) and organic synthesis (i.e. low water activity) require lipases to be active under extreme conditions, most lipases used are mesophilic. Many mesophilic lipases, which typically come from organisms like *Bacillus* and *Aspergillus* species, are active at high temperatures. As a result, extremophilic lipases are often overlooked; however, lipases from thermophilic *Bacillus* species have been shown to be more efficient than currently used enzymes ^53^.

## Glycosyl hydrolases and sugars

Glycosyl hydrolases hydrolyze the glycosidic bond between a carbohydrate and another moiety and are categorized into well over 100 families. The hydrolysis generally takes place with the use of only two amino acids—a proton donor and a nucleophile/base—and results in retention or inversion of the anomeric configuration of the resulting carbohydrate.

Roughly 70% of the world’s population ^54^ suffers from lactose intolerance resulting from a lack or loss of β-galactosidase activity. For this majority of the population, the best way to avoid the often embarrassing symptoms of lactose intolerance is through the consumption of lactose-free milk and other dairy products, which are generated via the use of the lactase (β-galactosidase) from organisms like *Kluyveromyces lactis* ^54^. However, for the enzyme to be active, the temperature of the dairy product must be raised (from about 5°C to 25°C). This elevation in temperature creates the potential for pathogens to grow as well as for altering the flavor profile of the milk. A simple solution to both issues is to use a β-galactosidase from a psychrophile ^1^. This enzyme would be active at low temperature and hydrolyze lactose throughout the entire process from production to shipment and storage by the consumer ^55^. This approach could save significant amounts of money by eliminating the heating step as well as achieve a high percentage of lactose hydrolysis. Currently, several cold-adapted enzymes have been characterized and developed that perform on par with the currently used mesophilic enzymes when compared at their respective temperature optima (i.e. 15°C and 37°C) ^1, 55, 56^.

Similar to the industrial-scale hydrolysis of lactose, that of starch traditionally uses mesophilic enzymes. Starch-hydrolyzing enzymes comprise about 25% of the worldwide enzyme market; however, several adjustments in temperature and pH are needed for most of the reactions to ensure optimal conditions.

Since the industrial processes involved in hydrolyzing starch require high temperatures (95°C for one step and 60°C for the other) and high pH, polyextremophilic (thermophilic and alkaliphilic) enzymes would be ideal. Currently, an α-amylase from *Bacillus acidicola* ^57^, glucoamylases from *Picrophilus* ^58^, and a pullulanase from *Thermococcus kodakarensis* ^59^ show great promise in replacing their mesophilic counterparts. However, amylases have also been isolated from halophiles, such as *Halomonas meridian* and *Natronococcus amylolyticus*, that could be useful in the process of producing high-fructose corn syrup, which is produced by hydrolyzing corn starch ^43^.

In addition to sugar hydrolysis, another promising application for extremophiles is the production of carbohydrates like trehalose and ectoine, which can be used as stabilizers for products like antibodies and vaccines ^60, 61^. The production of trehalose from *Sulfolobus solfataricus* in a bioreactor has been perfected and could easily replace the currently used mesophilic enzymes from *Arthrobacter* species Q36 ^43^. Another example is ectoine, which has been shown to protect skin from UVA-induced damage. RonaCare™ Ectoin, produced by Merck KGaA (Darmstadt, Germany) is used as a moisturizer and comes from halophilic microorganisms ^39^. In addition to trehalose and ectoine, several other carbohydrates are produced by halophiles as compatible solutes that can also be employed as preservatives ^62^.

## Medical applications

Surprisingly, microorganisms, including extremophiles, are producers of a host of antibiotics, antifungals, and antitumor molecules ^63^. In truth, this should come as little surprise, as microorganisms have been killing each other and fighting for survival for billions of years. After that long a time, it should be clear that microorganisms have perfected the art of warfare, but it is up to us to take advantage of it.

In addition to the typical antibiotics known from mesophilic microorganisms ^64, 65^, extremophiles are known to generate antimicrobial peptides and diketopiperazines ^66^. Antimicrobial peptides have been found in the Halobacteriaceae (phylogenetic family containing all halophilic archaea) as well as *Sulfolobus* species. These peptides (halocins) from halophilic archaea are thought to be found in all species of the family. Each halocin has a specific range of activity, and some act on a broader range of microorganisms than others ^39^. Halocins have been shown to be effective at killing archaeal cells; however, there are no data to show that halocins kill microorganisms pathogenic to humans. Interestingly, there is evidence that they assist canines in recovering from surgery ^67^.

Diketopiperazines (also known as cyclic dipeptides) have been shown to affect blood-clotting functions as well as having antimicrobial, antifungal, antiviral, and antitumor properties. They are found in halophiles like *Naloterrigena hispanica* and *Natronococcus occultus* ^45^ and have been shown to activate and inhibit quorum-sensing pathways ^66^. These pathways are important in pathogens such as *Pseudomonas aeruginosa*, which is one of the causative agents of pneumonia and a typical infection found in patients with cystic fibrosis ^68, 69^. Therefore, this could be an alternative treatment for the tens of thousands of drug-resistant *Pseudomonas aeruginosa* infections that occur each year ( http://www.cdc.gov/hai/organisms/pseudomonas.html).

In addition to molecules that kill other organisms and tissues, extremophiles can also play a role in the medical field through the use of bioplastics. Several species of extremophiles produce polyhydroxyalkanoates (PHAs), which are a heterogeneous group of polyesters; however, they are most commonly found in the halophilic archaea ^39^. For example, it has been shown that *Haloferax medeterrani* can be grown with 65% of its dry weight as PHAs, which translates into 6 g/L of culture when grown in media supplemented with starch ^45^. PHAs are often used as carbon storage for microbial cells but have been harnessed to generate bioplastics and have been lauded for their biocompatibility, resistance to water, and biodegrading properties, all of which make them an attractive alternative to petroleum-based plastics ^45^.

Finally, a very interesting extremophile contribution to the field of medicine comes in the form of an alternative vaccine delivery system ^70^. Several microorganisms produce internal gas vesicles, small gas-filled proteinaceous structures, the best-studied coming from the halophilic archaea. These structures have been engineered in *Halobacterium* species NRC-1 to generate a recombinant form that expresses portions of the simian immunodeficiency virus on the external surface ^71^. Once collected, these recombinant vesicles have shown a strong antibody response and immune memory when injected into mice. Typically, vaccines derived from recombinant methods require the addition of adjuvants (e.g. cholera toxin B) to elicit a large enough immune response ^71^. However, in the case of the recombinant gas vesicles from *Halobacterium* species NRC-1, the organism’s own polar lipids can be used as an adjuvant, as they raise a large immune response since they are ether linked as opposed to the more typical ester-linked molecules. Experiments using NRC-1’s polar lipids and recombinant gas vesicles as a nasal-delivered vaccine in mice were quite encouraging and showed no toxicity ^71^.

## Conclusions

With established commercial success in the DNA polymerase, biofuels, biomining, and carotenoid sectors of biotechnology, extremophiles and their enzymes have an extensive foothold in the market that is expected to keep growing. However, to fulfill this great potential, innovative methods will have to be developed to overcome current roadblocks. The most significant is a current lack of ability to produce most extremophiles/extremozymes on the large scale required by industrial processes. Some recombinant extremozymes can be produced in large quantities by mesophilic organisms like *Escherichia coli*; however, this is not true for most. Therefore, new expression systems will have to be developed with extremophilic organisms as the host to achieve high expression of soluble proteins. Another significant roadblock is the general lack of partnerships among academia, industry, and government. More opportunities for ties between all three groups should be encouraged, nurtured, and supported from all sides. For it is only with all three working together that the most progress will be made.

## Abbreviations

AMD, acid mine drainage; PCR, polymerase chain reaction; PHA, polyhydroxyalkanoate.
